# Tracking endothelium-dependent NO release in pressurized arteries

**DOI:** 10.3389/fphys.2023.1108943

**Published:** 2023-01-24

**Authors:** Lillian Wallis, Lucy Donovan, Aaron Johnston, Lauren C. Phillips, Jinheng Lin, Christopher J. Garland, Kim A. Dora

**Affiliations:** The Vascular Pharmacology Group, Department of Pharmacology, University of Oxford, Oxford, United Kingdom

**Keywords:** nitric oxide, fluorescent NO indicator, eNOS, acetylcholine, SNAP, DAR-4M AM, Cu_2_FL2E

## Abstract

**Background:** Endothelial cell (EC) dysfunction is an early hallmark of cardiovascular disease associated with the reduced bioavailability of nitric oxide (NO) resulting in over-constriction of arteries. Despite the clear need to assess NO availability, current techniques do not reliably allow this in intact arteries.

**Methods:** Confocal fluorescence microscopy was used to compare two NO-sensitive fluorescent dyes (NO-dyes), Cu_2_FL2E and DAR-4M AM, in both cell-free chambers and isolated, intact arteries. Intact rat mesenteric arteries were studied using pressure myography or *en face* imaging to visualize vascular smooth muscle cells (SMCs) and endothelial cells (ECs) under physiological conditions. Both NO-dyes irreversibly bind NO, so the time course of accumulated fluorescence during basal, EC-agonist (ACh, 1 µM), and NO donor (SNAP, 10 µM) responses were assessed and compared in all experimental conditions. To avoid motion artefact, we introduced the additional step of labelling the arterial elastin with AF-633 hydrazide (AF) and calculated the fluorescence ratio (FR) of NO-dye/elastin over time to provide data as FR/FR_0_.

**Results:** In cell-free chambers using either Cu_2_FL2E or DAR-4M AM, the addition of SNAP caused a time-dependent and significant increase in fluorescence compared to baseline. Next, using pressure myography we demonstrate that both Cu_2_FL2E and DAR-4M AM could be loaded into arterial cells, but found each also labelled the elastin. However, despite the use of different approaches and the clear observation of NO-dye in SMCs or ECs, we were unable to measure increases in fluorescence in response to either ACh or SNAP when cells were loaded with Cu_2_FL2E. We then turned our attention to DAR-4M AM and observed increases in FR/FR_0_ following stimulation with either ACh or SNAP. The addition of each agent evoked an accumulating, time-dependent, and statistically significant increase in fluorescence within 30 min compared to time controls. These experiments were repeated in the presence of L-NAME, an NO synthase inhibitor, which blocked the increase in fluorescence on addition of ACh but not to SNAP.

**Conclusion:** These data advance our understanding of vascular function and in the future will potentially allow us to establish whether ECs continuously release NO, even under basal conditions.

## 1 Introduction

Since its discovery and identification as an endothelium-derived relaxing factor, nitric oxide (NO) has been established as an important signalling molecule in the vasculature. Following synthesis, NO diffuses out of endothelial cells (ECs) past the components in the extracellular space and into adjacent vascular smooth muscle cells (SMCs) to stimulate vasodilation. NO is an unstable, highly lipophilic free radical, with a half-life of sub-seconds *in vivo*, but minutes in the absence of oxyhaemoglobin in buffered solution *in vitro* (Liu et al., 1998). It readily reacts with oxygen and haem-iron to yield more stable nitrite/nitrate compounds; therefore, accurate detection of NO in arteries is problematic. The various methods used previously to detect and quantify NO synthesis and release from arteries have been comprehensively reviewed (Yao et al., 2004; Moller et al., 2019). Some methods are indirect and cannot be used in living tissues such as the colorimetric Griess assay or the chemiluminescent luciferin-luciferase system. Other methods, including microelectrode NO sensing, offer rapid response times and good sensitivity but are limited by factors such as gas interference (Yao et al., 2004). An ISONOP30 electrode (length 0.5–2 mm, diameter 30–60 µm) with selectivity and high sensitivity for NO has been successfully utilized in intact arteries mounted under isometric conditions (Simonsen et al., 1999; Hernanz et al., 2004). However, although this technique may serve to confirm other approaches, it may not be possible to generate routine data in pressurized resistance arteries.

The use of NO-sensitive fluorescent dyes (NO-dyes) to measure NO generated intracellularly has been explored more recently with varying degrees of success. In all cases, the binding of NO to the NO-dye is not reversible; therefore, changes in the rate and magnitude of fluorescence are measured, and ideally compared to time courses in the presence of a NO synthase (NOS) inhibitor. The first fluorescent NO-dyes developed were diaminobenzene-based fluorophores such as diaminofluoresceins (DAFs) which produce a fluorescent signal when bound to NO. Although simple to use and sensitive to low concentrations of NO, their use has been criticized due to severe interference by Ca^2+^ (Broillet et al., 2001). This interference may reflect the rate and magnitude of NO released by the NO donors (Suzuki et al., 2001) and, as such, DAFs may well be better suited to measure NO produced by ECs (Kojima et al., 1998). Structurally related to DAFs, diaminorhodamine (DAR) dyes are another class of fluorescent NO-dyes. Rhodamine-based dyes are considered superior to fluorescein-based dyes as they are more photostable, and their signal is less influenced by artery autofluorescence (e.g., from elastin) due to the longer excitation wavelength. A cell-trappable DAR dye, DAR-4M AM, has been successfully used in cultured bovine aortic ECs (Kojima et al., 2001), but has not yet been validated in intact arterial preparations. Once inside the cells, DAR-4M AM is deacetylated by intracellular esterases to form DAR-4M, trapping it intracellularly. It is then converted to fluorescent DAR-4M triazole (DAR-4M T) by binding with NO (Kojima et al., 2001).

Additionally, membrane-permeable NO-dyes, such as copper-based fluorophores, are also available and have shown promise in the ability to detect NO levels. In comparison to DAF/DAR-based indicators, they detect NO directly and are non-toxic; however, previous protocols using copper-based fluorophores such as Cu_2_FL2E have required the use of high concentrations of both Cu^2+^ (McQuade et al., 2010) and DMSO (McQuade et al., 2010; Ghosh et al., 2013) which are both known to cause arterial dysfunction (Yi et al., 2017).

In this study, we draw on our expertise using fluorescent Ca^2+^-indicator dyes in arteries (Rodríguez-Rodríguez et al., 2009; Bagher et al., 2012; Garland et al., 2017) to characterize the fluorescent NO-dyes, Cu_2_FL2E and DAR-4M AM, and optimize their use in isolated rat resistance arteries mounted in a pressure myograph. We establish DAR-4M AM as a valuable tool for reliable relative quantification of NO production in intact arteries, which we hope may be utilized to elucidate further the role of NO in the regulation of vascular function.

## 2 Materials and methods

### 2.1 Animal and tissue preparation

Animal use was approved by the University of Oxford ethics committee and complied with the Animals (Scientific Procedures) Act 1986 and European Directive 201/63/EU. Animals were housed in a temperature-controlled environment in a 12-h light-dark cycle with food and water supplied *ad libitum.* Male Wistar rats (Charles River, United Kingdom; 200–300 g) were euthanized by inhalation of CO_2_ and cervical dislocation in accordance with Schedule 1 of the A(SP)A 1986, United Kingdom.

The mesenteric bed was removed and stored in ice-cold Krebs physiological solution (KPS) containing (mM): 121.3 NaCl, 25.0 NaHCO_3_, 11.0 glucose, 4.7 KCl, 2.5 CaCl_2_, 1.2 MgSO_4_·7H_2_O, 1.18 KH_2_PO_4_ and gassed with 21% O_2_, 5% CO_2_, 74% N_2_. Third-order rat mesenteric arteries were cleared of adherent tissue and isolated for further study.

**FIGURE 1:**
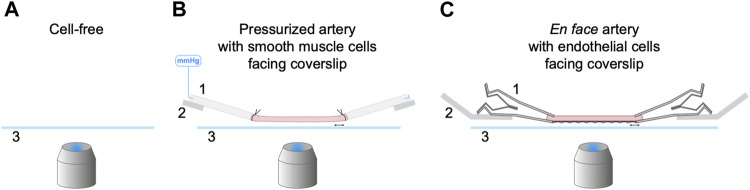
Three experimental setups for the characterization of NO-dyes. **(A)** Cell-free setup with no artery present. NO-dye was added to a 2 mL chamber, and fluorescence detected 200 µm above the coverslip. Both PBS and KPS were used at 25°C and KPS also at 37°C. **(B)** Isolated, cannulated and pressurized artery in KPS at 37°C, with SMCs facing the coverslip. **(C)** Isolated, opened and stretched artery in KPS at 37°C for *en face* imaging, with ECs facing the coverslip. 1, a securing device (pipette or micro clamp); 2, an arm to hold the securing device (micromanipulator or caliper) that can be adjusted laterally to stretch the artery held above 3, a coverslip; imaged using an inverted microscope.

In this study three approaches were used to characterize and optimize the use of NO-dyes. First, cell-free experiments were performed to establish whether the NO-dyes respond to NO donors while still esterified. This high-throughput method was also useful for establishing important steps in the use of the NO-dyes. Next, we used pressurized arteries. This approach was limited to adding the NO-dyes to the outside of arteries to image SMCs. Luminal pumping of the NO-dye into ECs would potentially activate endothelial NO synthase (eNOS) and, since the binding of NO was not reversible, this loading method was considered not to be reliable. Instead, to image ECs we used a third approach based on our recent work with cardiac myocyte strips (Borysova et al., 2021), whereby longitudinally-opened arteries were stretched between micro clamps to enable *en face* imaging of ECs (Figure 1).

Before NO-dye loading of intact arteries, elastin was labelled with 100 nM Alexa Fluor™ 633 hydrazide (AF; Invitrogen, MA, United States) added into the bath for 20 min prior to the experiment.

### 2.2 NO-dye preparation

In all experiments the working solution of NO-dye was freshly prepared immediately before use.

#### 2.2.1 Cu_2_FL2E preparation

FL2E (0.5 mg; 07-0291, STREM Chemicals, Cambridge, United Kingdom) was dissolved in DMSO to make a stock solution of 1.0 mM FL2E. This was aliquoted and stored at −80°C as per the product sheet. When thawed, the solution was kept chilled in the dark and used as quickly as technically feasible; any unused dye was discarded.

Note that each FL2E molecule has the potential to bind two copper ions (hence Cu_2_), and each of the copper ions can be displaced by NO to form FL2E-NO (or FL2A-NO inside cells once de-esterifed) and increase dye fluorescence. Therefore, when NO binds, Cu^2+^ becomes free in solution. We commenced studies using the working ratio of 1:2.

The steps in preparing Cu_2_FL2E (1 µM) for use in cell-free chambers were:

1. Prepare the working solution of Cu_2_FL2E (1 μM, working ratio 1:2) by adding 2 µL of stock (1 mM in DMSO) FL2E and 4 µL of stock (1 mM in H_2_O) CuCl_2_ to 2 mL buffered solution (either phosphate buffered saline (PBS) or KPS), at 25°C, mixing well.
2. In some experiments, higher concentrations of Cu_2_FL2E were used (2 μM and 5 µM).
3. In some experiments, the concentration of CuCl_2_ was increased to make working ratios of 1:5 and 1:10.

In experiments where Cu_2_FL2E was loaded into cells of arteries, additional steps were followed. We chose 2.5 µM FL2E as this maximized our ability to visualize NO-dye loading but also maintained levels of pluronic and DMSO lower than we have previously used for loading Ca^2+^-indicator dyes (Bagher et al., 2012).

The steps in preparing Cu_2_FL2E (2.5 µM) for use in arteries (already loaded with AF) were.

1. Prepare the working solution of Cu_2_FL2E (working ratio 1:2) by adding 5 µL of stock (1 mM in DMSO) FL2E and 10 µL of stock (1 mM in H_2_O) CuCl_2_ to 2 mL KPS, mixing well.
2. Add 3 µL pluronic™ F-127 (20% pluronic in DMSO; P3000MP, Invitrogen, MA, United States) to the 2 mL working solution, mixing well.
3. Remove the KPS from the chamber bathing the artery and immediately replace with 1 mL KPS working solution (2.5 µM Cu_2_FL2E; 0.03% pluronic, 0.37% DMSO). Ensure gassing is on, but note that the pluronic in the working solution will cause bubbles to form when it is gassed, so only part fill the chamber (1 mL in 1.5 mL chamber). Incubate for 20 min to load the artery.
4. Wash with KPS for >5 min to remove residual NO-dye.

#### 2.2.2 DAR-4M AM preparation

DAR-4M AM (1 mg; 251765, Calbiochem, CA, United States) was dissolved in DMSO to make a stock solution of 5.0 mM DAR-4M AM. This was aliquoted and stored at −20°C. When thawed, this solution was kept chilled in the dark and used as quickly as technically feasible.

The steps for preparing DAR-4M AM (5 µM) to use in cell-free chambers were:

1. Dilute the stock DAR-4M AM (5 mM) into buffered solution (PBS or KPS).

In experiments where DAR-4M AM was loaded into cells of intact arteries, additional steps were followed. We chose 5 µM DAR-4M AM as this allowed us to maximize the ability to visualize NO-dye loading as well as maintain levels of pluronic and DMSO similar to those used for Cu_2_FL2E.

The steps in preparing DAR-4M AM (5 µM) for use in arteries (already loaded with AF) were.

1. Prepare the working stock by adding 1 µL of stock (5 mM) DAR-4M AM to 3 µL pluronic™ F-127 in a 0.5 mL Eppendorf tube and triturate well.
2. Add the 4 µL of working stock to the chamber bathing the artery in KPS (1 mL). This working solution contains 5 µM DAR-4M AM, 0.06% pluronic, 0.34% DMSO.
3. Ensure gassing is on and incubate for 20 min to load the artery.
4. Wash with KPS for >5 min to remove residual NO-dye.

### 2.3 Cell-free experiments

The first set of experiments aimed to establish the extent of NO-dye fluorescence in response to the NO donor *S*-Nitroso-*N*-acetyl-DL-penicillamine (SNAP; Abcam, Cambridge, United Kingdom), and how varying experimental conditions impacted this response. These experiments were performed using a 2 mL chamber (RC-27; Warner Instruments, CT, United States) containing room temperature (∼25°C), buffered solution. The chamber was seated within the stage of an Olympus inverted microscope, as previously described using tissue (Garland et al., 2017). The solution was routinely visualized 200 µm above the coverslip using an Olympus 20x water immersion (0.17 NA, 0.70 mm WD) and linescan confocal microscope (FV300 or FV500) with FluoView software (Olympus, Tokyo, Japan). Each image was 512 × 512 pixels, equating to images of x = 318 µm by y = 318 µm. The frequency of acquiring images for each NO-dye was 0.45 Hz.

Each NO-dye was added to the chamber, mixed and allowed to equilibrate for at least 2 min. Once the signal was stable, SNAP was added, and acquisition continued for ∼15 min.

The acquisition settings remained consistent between data sets for each NO-dye. Cu_2_FL2E was excited at 488 nm, emitted light detected at ≥ 505 nm; DAR-4M AM was excited at 543 nm, emitted light detected at ≥ 560 nm.

### 2.4 Wire myography

Isolated arteries were mounted in a Mulvany-Halpern wire myograph chamber (model 610, Danish MyoTechnology, Denmark) as described previously (Smith et al., 2020). Artery viability was determined using the α_1_-adrenergic vasoconstrictor phenylephrine (PE, 3 μM; Sigma-Aldrich, MO, United States) to assess SMC function, followed by the EC-dependent agonist acetylcholine (ACh, 10–100 nM; Merck, NJ, United States) to assess EC function. Arteries which demonstrated robust vasoconstriction to PE and >95% vasorelaxation to ACh were considered viable. The effect of CuCl_2_ on arterial function was examined by incubating arteries with varying concentrations of Cu^2+^ (1–10 µM) for 30 min, and responses to 3 μM PE then 10–100 µM ACh repeated. Vasoconstriction is expressed as the increase in tension above baseline and was adjusted to account for length of each artery (mN/mm); relaxation is expressed as a percentage reversal of tone induced by PE. Only functional experiments were performed using wire myography.

### 2.5 Pressure myography

Isolated arteries (>1 mm length) without side-branches were transferred to a pressure myography chamber (1.5 mL; RC-27N, Warner Instruments, CT, United States) containing chilled KPS, held within the stage of an Olympus FV500 or FV1000 microscope. Arteries were then cannulated using glass micropipettes (maximum outer diameter 120 μm) and secured using 11/0 sutures (Ethicon, NJ, United States). The lumen was gently flushed to remove excess blood prior to cannulating the distal end.

The chamber was maintained at 36.6°C ± 1°C and gassed to maintain pH for the duration of the experiment. During experimental set-up, the chamber was continuously superfused with warmed KPS *via* an inlet tube and removed *via* an outlet tube at a rate of 1–1.5 mL/min. Arteries were pressurized to 70 mmHg to mimic physiological blood pressure using a gravity-fed pressure tower, whilst being stretched longitudinally until straight. Pressurized arteries were visualised using an Olympus FV500 linescan confocal microscope running FluoView software (Olympus, Tokyo, Japan). When studying function, pressurized arteries were imaged with transmitted light using a 10x Olympus (0.40 NA, 3.1 mm WD) objective. A leak test was performed by closing the tap to the pressure tower and measuring artery deflation for 1 min. Only arteries with minimal transmural leaks (<5% deflation in 1 min) were used further. The functional response of all leak-free arteries was assessed by observing contraction to 3 μM PE then vasodilation to ACh (10 nM–100 nM).

As the NO-dye protocol was being developed it was clear that measures were required to prevent movement artefacts. This was achieved by performing the NO-dye experiments in the continued presence of the L-type voltage-gated Ca^2+^ channel blocker nifedipine (1 μM; Sigma-Aldrich, MO, United States). As a second measure to avoid movement artefacts, arteries were incubated with the elastin label AF. Since the internal elastic lamina separating ECs and SMCs is very thin yet visible, and there is elastin between adjacent SMCs, this ‘template’ of artery structure could be captured by acquiring z-stacks. The same focal planes could then be selected for comparative images over time. This elastin label was also apparent with both NO-dyes, so movement and relative changes could both be assessed to improve the clarity of the NO-dye signals.

#### 2.5.1 NO-dye loading into arteries

Each NO-dye was added to the chamber, mixed, and allowed to load from the outside of arteries. Once the signal was clear above the background autofluorescence, the time course of baseline, 1 μM ACh, and 10 µM SNAP (each 30 min), was recorded in a static chamber with gassing. In some experiments no agonists were added (time control), and other experiments were performed in the presence of the NOS inhibitor L-NAME (100 μM; incubated for at least 20 min, Sigma-Aldrich, MO, United States).

The artery wall was imaged using a 40x (1.15 NA, 0.25 mm WD, Olympus) water immersion objective, obtaining 13.5 μm z-stacks in 1.5 μm steps. Each image plane was 780 × 340 pixels, equating to image volumes of x = 351 μm, y = 153 µm and z = 13.5 µm. The acquisition settings remained consistent between data sets for each NO-dye. AF was excited at 635 nm, emitted light detected ≥660 nm Cu_2_FL2E was excited at 488 nm, emitted light detected at 505–550 nm; DAR-4M AM was excited at 543 nm, emitted light detected at ≥ 560 nm. For each NO-dye z-stacks were acquired every 2 min, with 83.2 s rest time between each stack, 90 min per run. In all cases, each line was the average of two scans (line Kalman) and the wavelengths were captured sequentially. The transmitted light signal was also recorded.

Cu_2_FL2E (2.5 µM; working ratio 1:2) or DAR-4M AM (5 µM) were added to a static bath to load from the outside of arteries. Signal from AF was not detected in the wavelengths used for acquiring the NO-dyes (488 nm and 543 nm), and equally no NO-dye signal was visible at 635 nm, even during maximal responses to SNAP.

### 2.6 *En face* mounting

While pinned in the dissecting dish, cleaned arteries were partially cut transversely to reveal an opening in the lumen. Curved Vannas micro scissors (World Precision Instruments, FL, United States) were used to cut longitudinally through the wall along one side, leaving a flat, rectangular-shaped artery with ECs on one surface and adventitia on the other. The flat section of artery, around 1–2 mm in length, was cut away from the rest of the tissue and transferred to an imaging chamber (5 mL volume; Danish Myo Technology, Confocal Cardiac Myograph) as described previously (Borysova et al., 2021), containing chilled KPS. The artery was secured at each end by two micro clamps (Fine Science Tools, CA, United States) and stretched to resemble its physiological length *in vivo.*

#### 2.6.1 NO-dye loading into arteries between micro clamps

Arteries were loaded with AF and either NO-dye using the same concentrations and durations as stated above in Section 2.5.

### 2.7 Data analysis

Data were analysed using Microsoft Excel 2011 (v16, Microsoft Corporation, United States) and GraphPad Prism (v9.0, GraphPad Software, United States) software. All results are summarized as mean ± SEM of *n* replicates (unless otherwise stated), where *n* is the number of separate cell-free chamber experiments, or the number of individual arteries obtained from separate animals.

Normality was assessed using the Shapiro-Wilk test. If data were normally distributed, statistical testing was applied using a Student’s *t*-test, or one-way or repeated measures ANOVA, using multiple comparisons where appropriate. Statistical significance is defined as *p* < 0.05.

#### 2.7.1 NO-dye in the absence of cells

For data analysis of cell-free experiments, data were analyzed offline using MetaMorph software (version 7.7.4.0, Molecular Devices). The whole image field was defined as the region of interest (ROI), which was used to generate time courses of DAR-4M AM and Cu_2_FL2E fluorescence. Each fluorescence intensity value (F) was divided by the 30 s average before adding SNAP (F_0_), giving F/F_0_.

#### 2.7.2 NO-dye in arteries

For data analysis of the artery wall, data were first analyzed using Imaris software (version 8.0.2, Bitplane). The 13.5 µm z-stacks were visually inspected for the AF and NO-dye. The AF signal, indicating elastin, was an excellent indicator of artery movement. We noted that in all cases there was little movement of the artery, hence the AF signal did not vary over time. Once this was established, the 13.5 µm z-stack was merged into a single image plane, averaging the fluorescence for each time point and for each dye (wavelength). The files were saved as separate TIF files and opened in MetaMorph for further analysis. ROIs the size of the image field were used to generate separate time courses of fluorescence intensity for AF and each NO-dye. The fluorescence ratio (FR) of NO-dye/elastin over time was calculated to provide data as FR/FR_0._ For these experiments, the FR increased under baseline conditions; therefore, the value for FR_0_ was taken 30 min into each acquisition run, as the timepoint immediately prior to the addition of 1 µM ACh in other experiments. The FR/FR_0_ value was also expressed as a percentage of the fluorescence response to 10 µM SNAP, which represents maximum fluorescence in each experiment.

## 3 Results

### 3.1 Relaxation to SNAP and influence of Cu^2+^ on artery function

**FIGURE 2:**
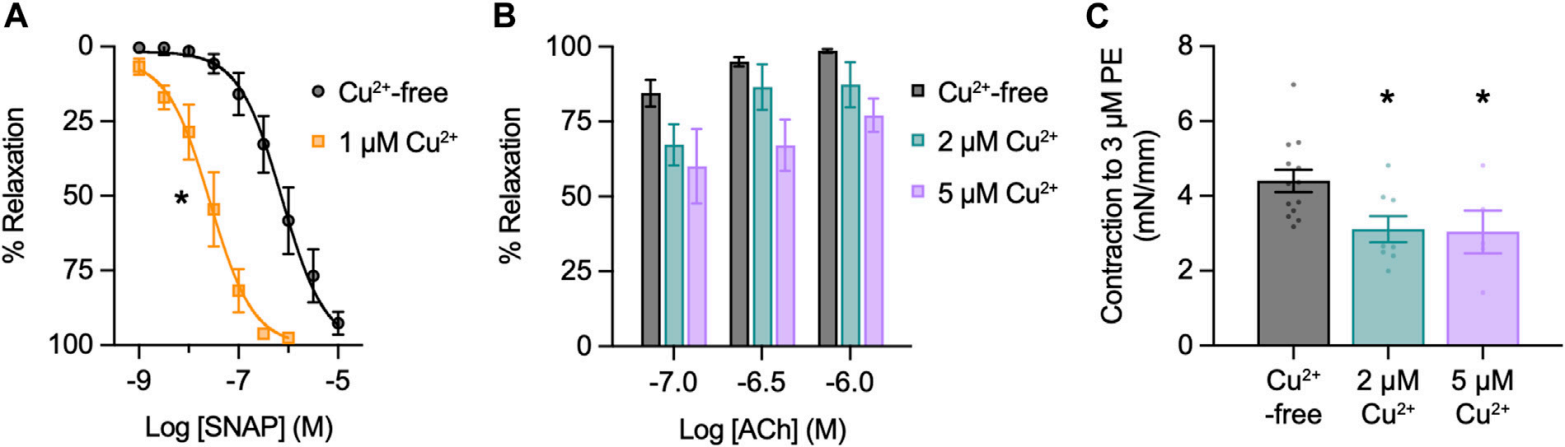
Effect of Cu^2+^ on artery function. Arteries were mounted in a wire myograph for measuring isometric tension. **(A)** Effect of 1 µM Cu^2+^ on concentration-dependent relaxation to SNAP in arteries contracted to PE. Non-parametric paired Wilcoxon test for log EC_50_ values; *, *p* = 0.031 vs. Cu^2+^-free (*n* = 6). **(B)** Effect of Cu^2+^ on concentration-dependent relaxation to ACh in arteries contracted to PE. Non-parametric unpaired KruskalWallis test with Dunn’s multiple comparisons; *p* = 0.742 and *p* = 0.051 in the presence of 2 μM and 5 µM Cu^2+^ vs. Cu^2+^-free, respectively (*n* = 4 for each). **(C)** Effect of Cu^2+^ on contraction to PE. Parametric paired *t*-test; *, *p* = 0.038 and *p* = 0.002 in the presence of 2 μM and 5 µM Cu^2+^ vs. Cu^2+^-free, (*n* = 8 and *n* = 5), respectively.

SNAP stimulated concentration-dependent relaxation of mesenteric arteries, with an EC_50_ of 0.74 µM and E_Max_ with 10 µM SNAP (Figure 2A). Therefore, to replicate functionally-relevant concentrations of SNAP, we used 10 µM SNAP to characterize the use of NO-dyes. Next, since Cu^2+^ is added to the FL2E solution and is displaced from Cu_2_FL2E to allow binding of NO, we characterized the influence of Cu^2+^ on mesenteric artery function. First, we established if Cu^2+^ influenced the response to SNAP. Preincubation with 1 µM Cu^2+^ left-shifted the SNAP concentration response curve (EC_50_ became 0.24 µM and E_Max_ was 1 μM, Figure 2A). Mechanistically, this is most likely attributable to the Cu^2+^-catalysed decomposition of *S*-nitrosothiols to yield NO (Dicks et al., 1996; Williams, 1999). By increasing the degree of SNAP decomposition, and thus NO generation, the presence of 1 µM Cu^2+^ augmented the relaxation to SNAP. Moreover, the rate of SNAP decomposition has been suggested to be proportional to the product of [Cu^2+^] and [*S*-nitrosothiol]; should this rate equation hold, it would explain the heightened rate of fluorescence accumulation. The effect of Cu^2+^ on EC and SMC function was then established. Since 2 μM and 5 µM Cu^2+^ were added to generate working ratios of 1:2 when using 1 μM and 2.5 µM Cu_2_FL2E, respectively, arteries were preincubated with 2 μM and 5 µM Cu^2+^ and the responses to ACh and PE compared to Cu^2+^-free conditions. Relaxation to ACh tended to be reduced by the presence of Cu^2+^, and Cu^2+^ significantly reduced contraction to PE (Figures 2B, C). At higher concentrations (10 µM), Cu^2+^ caused a slow but substantial increase in artery tone over 20 min followed by aberrant relaxation to ACh (data not shown). This indicates that elevated extracellular Cu^2+^ is damaging to arteries and as such, levels of exposure need to be minimized.

In light of this, [Cu^2+^] was kept to a minimum in live-cell experiments. Furthermore, care was taken when considering responses to SNAP in the presence of Cu_2_FL2E. The Cu^2+^ displaced by NO would potentially be available to augment the release of NO from SNAP, establishing a positive feed-forward loop resulting in increased NO-dye fluorescence.

### 3.2 Response of Cu_2_FL2E and DAR-4M AM to SNAP in cell-free chambers

The first step in validating the use of Cu_2_FL2E and DAR-4M AM was to test their response to 10 µM SNAP in cell-free chambers containing PBS.

#### 3.2.1 Cu_2_FL2E in the absence of cells

**FIGURE 3:**
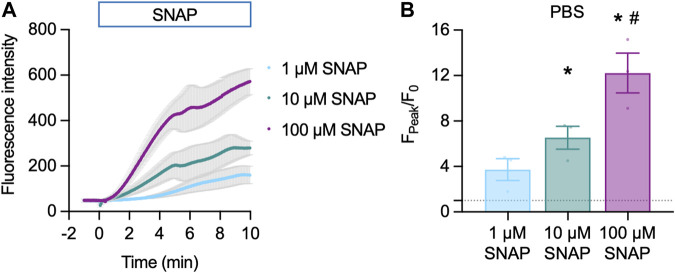
Cu_2_FL2E response to SNAP in the cell-free setup. Time course **(A)** and summary **(B)** of concentration-dependent increases in Cu_2_FL2E fluorescence in response to SNAP. Cu_2_FL2E was used at 1 μM, working ratio 1:2 in PBS at 25°C. SNAP was added to the chamber at *t* = 0 min. Repeated measures ANOVA with Tukey’s multiple comparisons test (*n* = 3 for all experiments); *, *p* < 0.0001 and *p* = 0.022 for 10 μM and 100 µM vs. 1 µM SNAP, respectively; ^#^, *p* = 0.047 vs. 10 µM SNAP.

**FIGURE 4:**
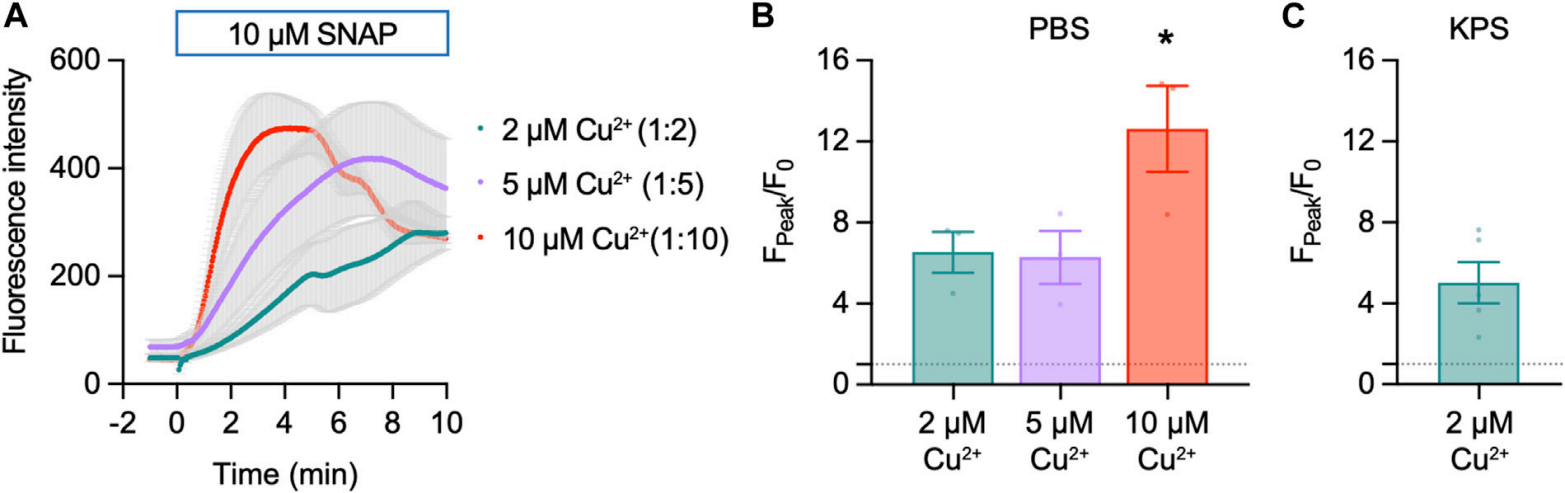
Effect of Cu^2+^ on Cu_2_FL2E response to SNAP in the cell-free setup. Time course **(A)** and summary **(B)** of concentration-dependent increases in Cu_2_FL2E fluorescence in response to 10 µM SNAP. Cu_2_FL2E was used at 1 μM, working ratios 1:2, 1:5, and 1:10 in PBS at 25°C. SNAP was added to the chamber at *t* = 0 min. Parametric paired *t*-test (*n* = 3); *, *p* = 0.031 vs. 2 µM Cu^2+^. **(C)** Summary of response to 10 µM SNAP when gassed KPS was used (*n* = 4–6).

Cu_2_FL2E (1 µM) was intrinsically fluorescent and addition of SNAP concentration-dependently increased NO-dye fluorescence, both in rate of increase and magnitude (Figure 3A). A 6.5 ± 1.0-fold (*n* = 3) increase in fluorescence was observed to 10 µM SNAP (Figure 3B). To establish whether a working ratio of 1:2 was limiting for fluorescence, it was increased to 1:5 and 1:10 (Figure 4A). No difference was observed at a working ratio of 1:5 (Figure 4B), suggesting an optimal working ratio of 1:2 for NO-dye fluorescence. This had the advantage of limiting the levels of free and potentially damaging [Cu^2+^]. Similar Cu_2_FL2E responses to 10 µM SNAP were observed in gassed KPS (5.0 ± 1.0-fold increase, *n* = 5, Figure 4C). The increased response to SNAP in the presence of 10 µM Cu^2+^ could be explained by an increase in the relative amount of FL2E bound to Cu^2+^ or an increase in the amount of NO generated from SNAP.

#### 3.2.2 DAR-4M AM in the absence of cells

**FIGURE 5:**
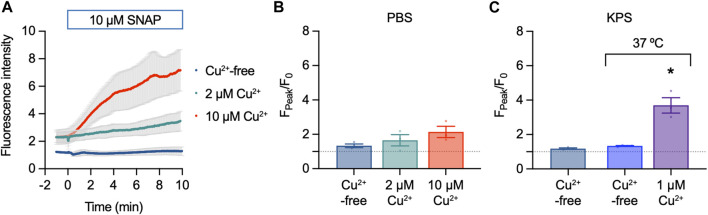
DAR-4M AM response to SNAP in the cell-free setup. **(A,B)** Effect of 10 µM SNAP and Cu^2+^ on DAR-4M AM fluorescence. DAR-4M AM was used at 5 μM, in PBS at 25°C. Ordinary one-way ANOVA; *p* = 0.8390 and *p* = 0.2382 in the presence of 2 μM and 5 µM Cu^2+^ vs. Cu^2+^-free (*n* = 3). **(C)** Effect of 10 µM SNAP and Cu^2+^ on DAR-4M AM fluorescence in gassed KPS at either 25°C or 37°C. In all experiments SNAP was added to the chamber for 10 min. Ordinary one-way ANOVA; *p* = 0.884 at 37°C vs. 25°C; *, at 37°C in the presence of 1 µM Cu^2+^ vs. Cu^2+^-free.

DAR-4M AM (5 µM) was also intrinsically fluorescent, however, addition of SNAP had little effect on NO-dye fluorescence (Figure 5A). A small 1.3 ± 0.1-fold (*n* = 3) increase in fluorescence was observed to 10 µM SNAP (Figure 5B). Raising [Cu^2+^] increased the fluorescence response of DAR-4M AM to 10 µM SNAP (Figures 5A, B), indicating the effect of Cu^2+^ facilitates the release of NO from SNAP. A similar effect was also observed in gassed KPS at 37°C, where 1 µM Cu^2+^ enabled the NO-dye to respond to 10 µM SNAP (Figure 5C).

### 3.3 Response of Cu_2_FL2E and DAR-4M AM to ACh and SNAP in pressurized arteries

After confirming that both Cu_2_FL2E and DAR-4M AM can respond to NO released from SNAP, we loaded each dye into pressurized rat mesenteric arteries.

#### 3.3.1 Cu_2_FL2E in pressurized arteries

**FIGURE 6:**
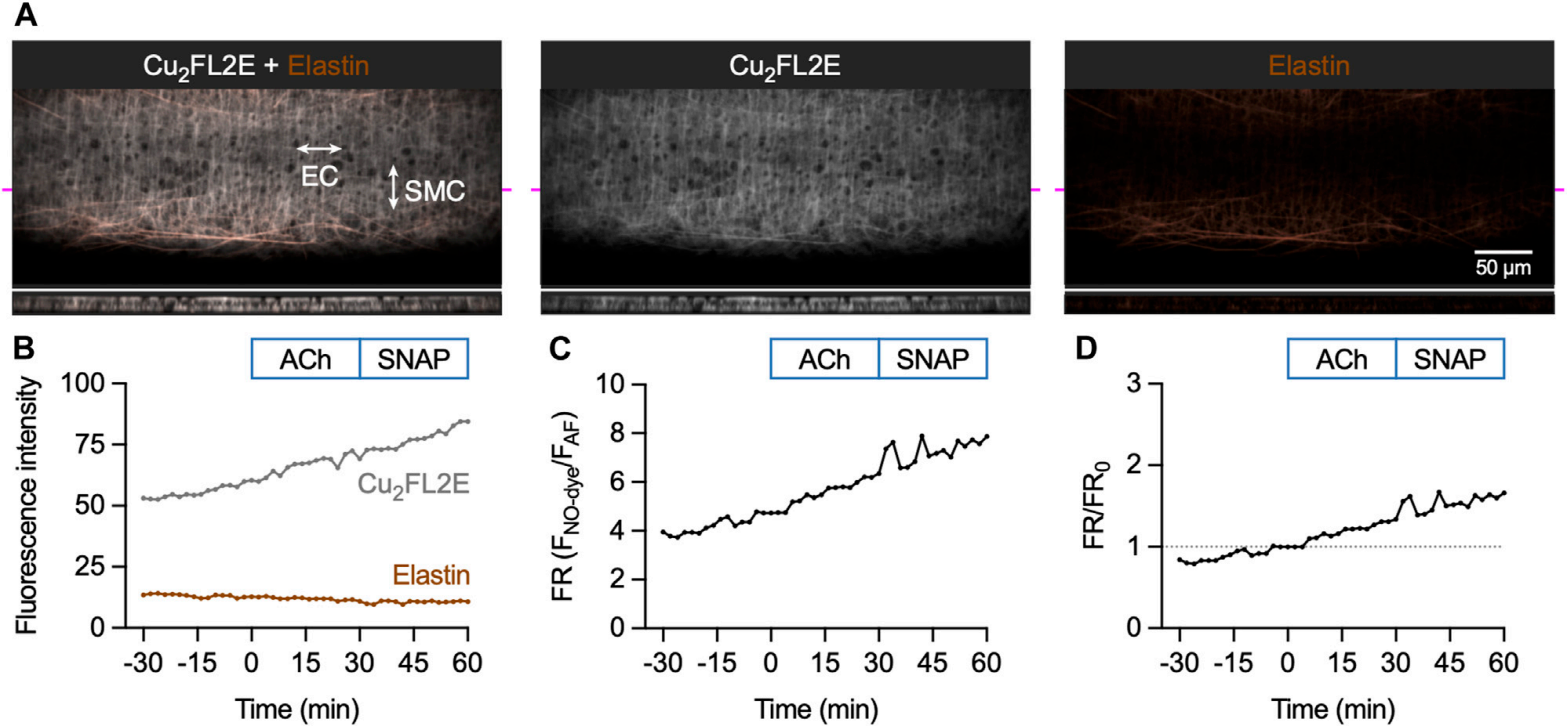
Cu_2_FL2E response to ACh and SNAP in the pressurized artery setup. **(A)** Representative confocal micrographs illustrating the ability of Cu_2_FL2E to load arteries. While the NO-dye clearly labelled elastin (black holes visible in IEL), SMCs were often visible. Cu_2_FL2E was used at 2.5 µM, working ratio 1:2 in gassed KPS at 37°C. The elastin was separately labelled with Alexa Fluor^®^ 633 hydrazide (AF, 10 nM). The bottom panels are cross sections through the artery wall at point indicated by magenta line. Merged z-stacks 13.5 µm thick into an x-y image. **(B)** Time course of changes in NO-dye and AF fluorescence following addition of 1 µM ACh at *t* = 0 min and 10 µM SNAP at 30 min (Supplementary Movie S1); representative of >5 experiments with slightly modified loading protocols. Time courses are also expressed as either a fluorescence ratio (FR) **(C)** or FR/FR_0_ (where *t* = 0 is always 1, dashed line) **(D)**. There was no clear response above the steady, gradual rise in Cu_2_FL2E fluorescence observed. Refer to Figure 7 for comparison.

Loading mesenteric arteries with AF and Cu_2_FL2E resulted in strong labelling of the arterial elastin with both dyes, as well as labelling the arterial SMCs (Figure 6A). Importantly, before loading the dyes there was almost no autofluorescence, but during NO-dye loading the internal elastic lamina (IEL) in particular displayed intense labelling. This provided a good indication that the dye had passed through all the SMC layers, but also meant that a large percentage of the fluorescence signal came from the elastin. Furthermore, it was essential that the NO-dye was thoroughly washed away from the artery, as any residual NO-dye in the chamber would respond to SNAP, as shown in the cell-free chambers (Figures 3, 4).

The response to activation of eNOS was then assessed by adding the EC-dependent vasodilator ACh, followed by exposure to the NO donor SNAP. The 90 min time course for Cu_2_FL2E-loaded arteries shows a constant, gradual increase in NO-dye fluorescence and a relatively stable level of AF fluorescence (Figure 6B; Supplementary Movie S1). The rate of increase remained unchanged over this time course and was not augmented by addition of either 1 µM ACh or 10 µM SNAP (Figures 6B–D). The increase in NO-dye, but not AF, fluorescence intensity and a lack of clear response to ACh or SNAP suggests that caution must be exercised when using this dye in intact arteries and indicated that Cu_2_FL2E is not suitable for use in pressurized mesenteric artery experiments.

#### 3.3.2 DAR-4M AM in pressurized arteries

**FIGURE 7:**
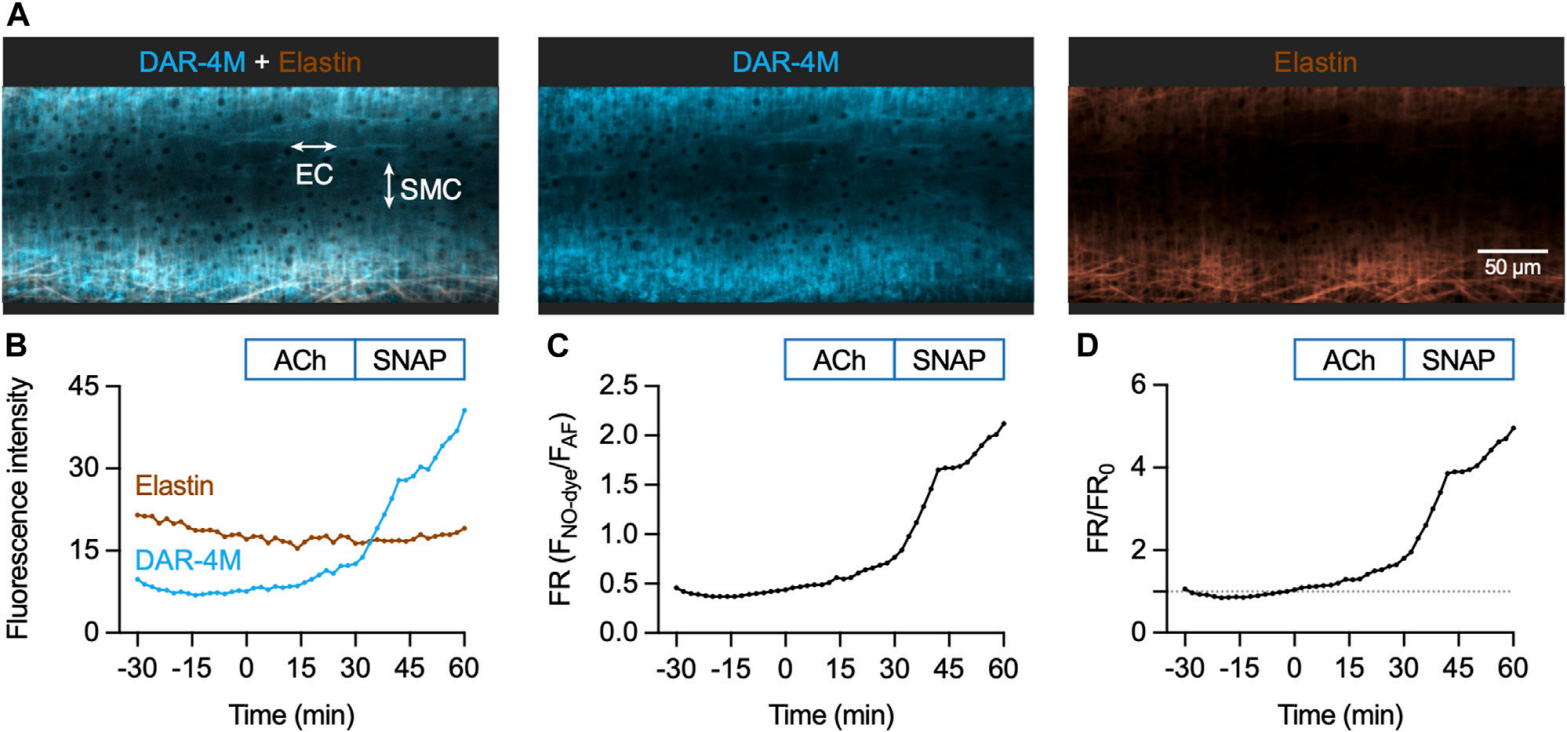
DAR-4M AM response to ACh and SNAP in the pressurized artery setup. **(A)** Representative confocal micrographs illustrating the ability of DAR-4M AM to load arteries. While the NO-dye clearly labelled elastin (black holes visible in IEL), SMCs were often visible. DAR-4M AM was used at 5 μM, in gassed KPS at 37°C. The elastin was separately labelled with Alexa Fluor^®^ 633 hydrazide (AF, 10 nM). Merged z-stacks 13.5 µm thick into an x-y image. **(B)** Time course of changes in NO-dye and AF fluorescence following addition of 1 µM ACh at *t* = 0 min and 10 µM SNAP at 30 min (Supplementary Movie S2). Time courses are also expressed as either a fluorescence ratio (FR) **(C)** or FR/FR_0_ (where *t* = 0 is always 1, dashed line) **(D)**.

In contrast to Cu_2_FL2E, loading of DAR-4M AM into mesenteric arteries enabled the detection of an increase in fluorescence in response to both endogenous and exogenous NO. Loading mesenteric arteries with AF and DAR-4M AM resulted in strong arterial elastin label by both dyes, and the NO-dye also labelled SMCs (Figure 7A). Importantly, before loading the dyes there was no detectible autofluorescence, and as with Cu_2_FL2E, during NO-dye loading the internal elastic lamina (IEL) was intensely labelled. This again was used as a strong indication that the dye had passed through all the SMC layers, but also meant that a large percentage of the fluorescence signal came from the elastin. As previously described, the NO-dye was thoroughly washed away from the artery, although the likelihood of signal from extra-arterial NO-dye in the chamber was low with DAR-4M AM (Figure 5).

The sequence of DAR-4M responses was matched to the Cu_2_FL2E dataset. As with Cu_2_FL2E, there was a constant, gradual increase in NO-dye fluorescence and a relatively stable level of AF fluorescence (Figure 7B; Supplementary Movie S2). Note that at the start of the protocol (∼15 min) a slight dip in NO-dye fluorescence was consistently observed, which may represent active pumping of DAR-4M out of SMCs. The fluorescence ratio is shown as raw data in Figure 7C, and compared to *t* = 0 min in Figure 7D, the latter enabling better comparisons between arteries.

**FIGURE 8:**
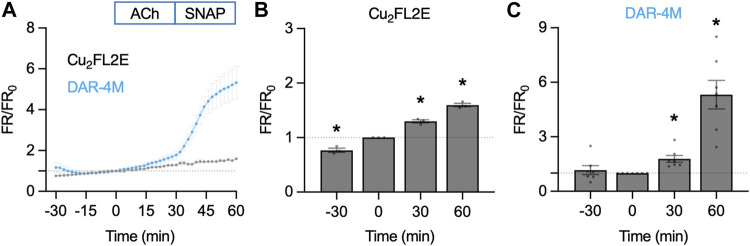
NO-dye response to ACh and SNAP in the pressurized artery setup. Comparison of summary data for each NO-dye. In all experiments 1 µM ACh was added at *t* = 0 min, and 10 µM SNAP at 30 min. **(A)** Summary data expressed as FR/FR_0_ for Cu_2_FL2E (*n* = 3) and DAR-4M (*n* = 7) loaded arteries. **(B)** Summary data for Cu_2_FL2E. Repeated measures one-way ANOVA with Dunnett’s multiple comparisons test; *, *p* = 0.050, *p* = 0.019, *p* = 0.006 for *t* = −30 min, 30 min, and 60 min vs. 0 min, respectively. **(C)** Summary data for DAR-4M. Repeated measures one-way ANOVA with Dunnett’s multiple comparisons test; *p* = 0.838, *, *p* = 0.017, and *, *p* = 0.004 for *t* = −30 min, 30 min, and 60 min vs. 0 min, respectively.

**FIGURE 9:**
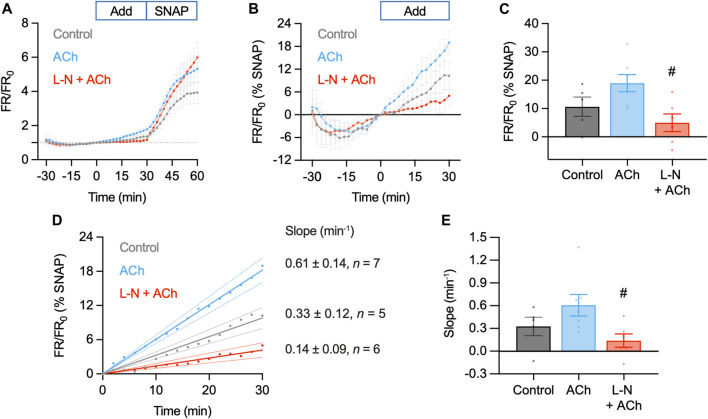
Summary of DAR-4M AM response to ACh and SNAP in the pressurized artery setup. In these experiments responses to 1 µM ACh (*n* = 7) were compared to control (basal accumulation without ACh, *n* = 5) and 1 µM ACh in the presence of L-NAME (L-N, 100 μM, *n* = 6), each added at *t* = 0 min. In all experiments 10 µM SNAP was added at 30 min. **(A)** Summary data expressed as FR/FR_0_ **(A)** and then as a percentage of the response to SNAP to better normalize the response between arteries **(B)**. Values at 30 min are summarized in **(C)**. Ordinary one-way ANOVA with Tukey’s multiple comparisons test; *p* = 0.238 for ACh vs. control; *p* = 0.576 and ^#^, *p* = 0.017 vs. L-N + ACh. Add, addition of either nothing (control), ACh or L-N and ACh. **(D)** Linear regression of individual experiments were averaged to obtain an index of the rate of NO release over the *t* = 0–30 min period (min^−1^); shown with 95% confidence intervals. Values are summarized in **(E).** Ordinary one-way ANOVA with Tukey’s multiple comparisons test; *p* = 0.286 for ACh vs. control; *p* = 0.575 and ^#^, *p* = 0.036 vs. L-N + ACh.

When directly compared, the summary time courses for each NO-dye are dissimilar (Figure 8). While in Cu_2_FL2E-loaded arteries there was a significant increase to both 1 µM ACh and 10 µM SNAP, these increases did not reflect any increase beyond the gradual basal increase, and were of small magnitude (Figures 8A, B). In contrast, the response to SNAP was clear in DAR-4M-loaded arteries (5.3 ± 0.8 fold increase in fluorescence ratio at 60 min compared to 0 min, Figures 8A, C). The response to 1 µM ACh was also significant as reported by DAR-4M (1.8 ± 0.2 fold increase at 30 min compared to 0 min), but to overcome possible gradual increases in baseline FR/FR_0_ we decided to compare to time controls (where no ACh was added), and went on to establish whether the responses were sensitive to block of NOS with L-NAME (Figure 9).

Although responses to ACh were not different to the control increase in FR/FR_0_, this can at least partly be accounted for by the apparent basal release of NO from arteries. Addition of L-NAME not only prevented the increase in FR/FR_0_ to ACh, but importantly also reduced the baseline increase (0–30 min, Figure 9A). Since the response to SNAP varied between arteries and data sets, data were normalized as a percentage of the SNAP response. This additional analysis step made comparisons between arteries more reliable. In so doing, it became clear that the release of NO from arteries (combined basal and ACh) was significantly reduced by L-NAME (Figures 9B, C). The same pattern emerged when reporting the rate of fluorescence ratio increase, an index of the rate of NO release (Figures 9D, E). This supports the use of DAR-4M AM in pressurized arteries, but it is clear that care must be taken when designing experiments and when interpreting data. When used in this way, DAR-4M can measure NO release, but approaches need to be improved to more reliably detect the release of NO in the full concentration range where it is able to stimulate vasodilation.

### 3.4 Response of DAR-4M AM and Cu_2_FL2E to ACh and SNAP in *en face* arteries

#### 3.4.1 DAR-4M AM and Cu_2_FL2E in *en face* arteries

**FIGURE 10:**
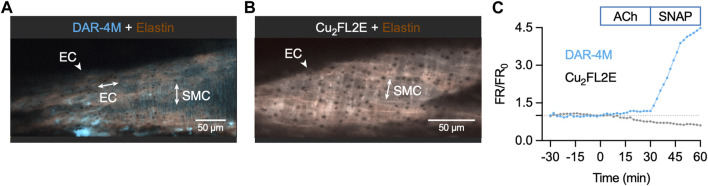
NO-dye response to ACh and SNAP in the *en face* artery setup. Representative confocal micrographs illustrating the ability of DAR-4M AM (5 µM) **(A)** and Cu_2_FL2E (2.5 µM, working ratio 1:2) **(B)** to load arteries in gassed KPS at 37°C. The elastin was separately labelled with Alexa Fluor^®^ 633 hydrazide (AF, 10 nM). Merged z-stacks 13.5 µm thick into an x-y image. **(C)** Time course of changes in NO-dye and AF fluorescence following addition of 1 µM ACh at *t* = 0 min and 10 µM SNAP at 30 min. This protocol was repeated at least 3 times.

**FIGURE 11:**
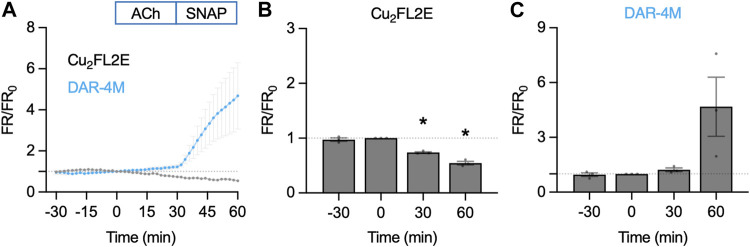
NO-dye response to ACh and SNAP in the *en face* artery setup. Comparison of summary data for each NO-dye. In all experiments 1 µM ACh was added at *t* = 0 min, and 10 µM SNAP at 30 min. **(A)** Summary data expressed as FR/FR_0_ for Cu_2_FL2E (*n* = 3) and DAR-4M (*n* = 3) loaded arteries. **(B)** Summary data for Cu_2_FL2E. Repeated measures one-way ANOVA with Dunnett’s multiple comparisons test; *p* = 0.758, *, *p* = 0.006 and *p* = 0.010 for *t* = −30 min, 30 min, and 60 min vs. 0 min, respectively. **(C)** Summary data for DAR-4M. Repeated measures one-way ANOVA with Dunnett’s multiple comparisons test; *p* = 0.952, *p* = 0.277, and *p* = 0.279 for *t* = −30 min, 30 min, and 60 min vs. 0 min, respectively.

To ensure the insensitivity of Cu_2_FL2E to NO was a result of the dye rather than ineffective cell loading, we attempted an alternative method of imaging intact arteries. Arteries were cut longitudinally to expose the ECs and mounted in a custom-made imaging chamber, allowing ECs and SMCs to be imaged with the ECs *en face* (Figure 10). Following the same sequence of NO-dye responses as in pressurized arteries, it was clear that even with visible ECs, the Cu_2_FL2E was unresponsive to either ACh or SNAP, yet DAR-4M was (Figures 10C, 11). While this confirmed that both indicators could be loaded into ECs and SMCs, we concluded that intracellular or elastin-bound Cu_2_FL2E was not responding to increased (NO) and this NO-dye should not be used in future experiments.

## 4 Discussion

The present study proposes a simple and reproducible protocol for the use of a NO indicator, DAR-4M AM, in *ex vivo* arteries. Our comparison of DAR-4M AM with Cu_2_FL2E in both cell-free chambers and live mesenteric arteries illustrates the importance of validating the characteristics of the fluorescent NO-dye used. We found that while both DAR-4M AM and Cu_2_FL2E responded to NO generated extracellularly from SNAP, only DAR-4M responded to endogenous NO production and NO donors within intact arteries.

Although there are many experimental NO donors available, we chose SNAP because of its widespread use in research associated with well-characterized temporal release profile (Feelisch, 1991; Simonsen et al., 1999; Ouyang et al., 2008; Ghosh et al., 2013). SNAP releases NO at a controlled rate, with one estimation claiming 1.4 µM NO is released from 100 µM SNAP per min, in PBS at 37°C and pH 7.4 (Feelisch, 1991). The relationship between (SNAP) and (NO) is complicated; NO release kinetics are highly sensitive to factors including transition metal ions (e.g., Cu^2+^), free thiols, pH and redox products (He and Frost, 2016). As such, it is difficult accurately to quantify the amount of NO produced from 10 µM SNAP in our experiments. Nevertheless, we chose 10 µM SNAP as a positive control as it produced maximal vasodilation in wire-mounted rat mesenteric arteries and corresponds to physiological [NO] reported in arteries (Simonsen et al., 1999; Hernanz et al., 2004). Simonsen et al. (1999) used microelectrodes to record NO production in wire-mounted rat mesenteric arteries and found that 10 µM SNAP caused relaxation and yielded a maximum NO concentration of 19 nM.

The use of NO-donors to characterize Cu_2_FL2E in cell-free systems is established (McQuade et al., 2010; Ghosh et al., 2013) and our data support these findings. The ability of Cu^2+^ to enhance the responses to SNAP is of interest when using this dye as both the working ratio of FL2E to Cu^2+^ and the Cu^2+^ freed upon binding of NO to FL2E will affect the final magnitude of response to SNAP. Interestingly while the response to SNAP is clear, the nitroxyl-donor Angeli’s salt did not affect Cu_2_FL2E fluorescence (McQuade et al., 2010), suggesting Cu_2_FL2E is selective for NO•. Responses to DAR-4M in cell-free preparations are also characterized (Kojima et al., 2001; Lacza et al., 2005). This dye is also not responsive to Angeli’s salt (Lacza et al., 2005), suggesting DAR-4M is also selective for NO•. In contrast, the use of NO-donors to characterize DAR-4M AM has not been demonstrated. The binding site for NO is distinct from the acetoxymethyl ester groups (Kojima et al., 2001) and hence, in theory, NO should still bind the AM form of the dye, yet how this affects fluorescence has not been defined. Our data suggest the AM groups may reduce responses to SNAP. Interestingly, addition of Cu^2+^ improved SNAP responses, which could reflect increased NO release from SNAP or a modification of the esterified dye to improve fluorescence on binding NO.

When used in cells previously, Cu_2_FL2E was able to respond to agonists. In the original study by McQuade *et al.* isolated cells and olfactory bulb slices responded to applied NO-producing agents, when using 1–5 µM Cu_2_FL2E at a working ratio of 1:2 with Cu^2+^ (McQuade et al., 2010). Moving to arterial cells, Ghosh *et al.* used 20 µM Cu_2_FL2E (working ratio 1:2) to load ECs in culture and reported a ∼ 4-fold increase in fluorescence in response to 10 μM ACh, which was effectively abolished by 100 μM L-NAME (Ghosh et al., 2013). In the same study, again using 20 μM, Cu_2_FL2E clearly loaded mouse carotid artery ECs and SMCs, and 10 µM ACh dilated the arteries and increased the fluorescence signal. The relaxation and fluorescence increase were both blocked by L-NAME. While these data seem convincing, this high concentration of Cu_2_FL2E (20 µM) and Cu^2+^ (40 µM) cannot be used in rat mesenteric arteries for multiple reasons. First, the high concentration of DMSO and pluronic used would activate EC Ca^2+^ and hence eNOS, during the loading protocol. We specifically used the lowest concentration of DMSO possible (<0.4%), as high concentrations of DMSO increase mesenteric artery EC Ca^2+^. This is especially relevant when using NO-dyes since the binding of NO to the dye is not reversible. Therefore, in our hands using 8-times more DMSO was not feasible, but we do appreciate that different vascular beds may have different sensitivities to DMSO. Second, the use of 40 µM Cu^2+^ is highly likely to cause arterial damage. Even under the assumption that all Cu^2+^ is bound to FL2E at the start of experiments (working ratio 1:2), as soon as NO displaces Cu^2+^ from FL2E the levels of Cu^2+^ could rapidly increase beyond 5 µM which we have shown is toxic to both ECs and SMCs. Our observation that Cu^2+^ impairs the function of wire-mounted rat mesenteric arteries remains unexplained but may reflect a pro-oxidant effect, resulting in the generation of harmful free radicals. Finally, our decision not to use Cu_2_FL2E in rat mesenteric arteries followed the observation that Cu_2_FL2E generated a very clear and intense label of elastin, and while this signal increased over time, it was not enhanced further by either ACh or SNAP. We suggest the binding of Cu_2_FL2E to elastin likely interferes with the ability of Cu_2_FL2E to bind NO. This is in addition to the fundamental failing of not detecting any response to either ACh or SNAP within loaded ECs or SMCs. These data suggest the use of Cu_2_FL2E in pressurized resistance arteries is not a reliable way to assess NO generation by ECs.

Our data indicate that DAR-4M AM is suitable to detect NO production by ECs in healthy mesenteric arteries. The rate of increase in fluorescence was slow compared to 10 µM SNAP, but both the basal release and that stimulated by 1 µM ACh could be detected. The NO detection threshold of DAR-4M AM is reported as 7 nM (Kojima et al., 2001), suggesting the NO-dye should be sufficiently sensitive to detect physiological NO in the nanomolar range. Moreover, our finding that L-NAME blocks the increase in DAR-4M (DAR-4M T) fluorescence following ACh addition suggests that the indicator is specific in detecting NO. It is important to note that DAR-4M does not directly bind to NO, and instead binds to NO^+^ equivalents such as nitric anhydride (Kojima et al., 2001). These equivalents are produced from the rapid auto-oxidation of NO; therefore, the NO-dye can only indirectly reflect NO levels. Nevertheless, the response to SNAP was marked, more so than in the cell-free chambers. This may reflect intracellular Cu^2+^ more effectively releasing NO from SNAP and/or a supra-additive effect of SNAP with other reactive oxygen species (Lacza et al., 2005), many of which would be present within cells. The ability to use this NO-dye in arteries was our initial aim, and an enhanced response with cells present is an advantage. It does, however, underline the importance of checking cell viability and keeping experimental conditions as consistent as possible (e.g., laser intensity, dye loading, pH), as cellular damage (as seen in many vascular diseases) is associated with increased levels of reactive oxygen species.

Finally, there is scope to further optimize the method presented above and ultimately enable the use of DAR-4M AM in other vascular beds. First, minimizing movement artefacts will allow (NO) to be compared within different arterial cell types. Our method allows ECs to be distinguished from SMCs in both pressurized and *en face* preparations. However, the movement of arteries during imaging makes measuring fluorescence within specific cells a challenge. All artery experiments were conducted in the presence of 1 µM nifedipine to minimize movement artefacts. Nifedipine cannot be used in experiments to establish whether basal NO release occurs normally in arteries with myogenic tone or during vasoconstriction. By blocking SMC VGCCs, nifedipine would prevent indirect activation of ECs by increases in SMC Ca^2+^ (Garland et al., 2017), signaling that may contribute to or even explain the basal release of NO. Instead, alternative approaches such as midplane imaging using 2 photon lasers (Bagher et al., 2012; Garland et al., 2017) could be characterized, retaining the use of AF to indicate the elastin signal. This method could then be extended to elucidate mechanisms of NO production in myogenic coronary and cerebral arteries which contract to L-NAME (Szekeres et al., 2004; McNeish et al., 2005). Investigating the fundamental properties of artery function, including NO production, is essential in understanding the function of healthy arteries and how this is altered by disease. As such, the technique we describe here provides potential to explore different aspects of vascular functionality.

For the visualization of ECs in this study we used an *en face* method for continuous live-cell imaging in arteries. Although this preparation is not as physiological as pressure myography, *en face* mounting offers many benefits. It allows arteries to be quickly and easily mounted, without the need to identify side branches during dissection, and allows homogenous populations of ECs or SMCs to be visualized clearly at high magnification. This makes the *en face* method of mounting arteries a valuable tool for use with multiple fluorescent indicators.

### 4.1 Study limitations

There is clearly much room for criticism and improvement of this methodology. The one-way nature of reporting NO levels with NO-dyes is far from ideal, especially compared to fluorescent Ca^2+^-indicator dyes that can report dynamic and subcellular changes in Ca^2+^ events. The development of a NO-dye that can release and re-bind NO to provide a dynamic signal would offer considerable advantage. Furthermore, with any NO-dye, an ability to improve the detection limit by ∼10-fold would be advantageous, particularly to enable basal NO release to be assessed. Finally, a cell-loadable NO-dye that does not bind elastin and can be dissolved in lower concentrations of DMSO would open new avenues for reporting EC levels of NO in pressurized arteries. The ability to luminally pump the dye to selectively image ECs in pressurized arteries, as routinely performed by our group when studying EC Ca^2+^ (Dora et al., 1997; Rodríguez-Rodríguez et al., 2009; Bagher et al., 2012; Garland et al., 2017; Dora et al., 2022a; Dora et al., 2022b), would be of great benefit. Using the *en face* preparation has advantages for studying ECs in arteries without the need for luminal pumping, but our approach is not yet optimized and requires further development.

Another consideration is that DAR-4M is limited to measuring NO• and does not report EC release of nitroxyl (HNO). Although NO• is released from mesenteric artery ECs, we and others have shown that nitroxyl can also be released by ACh (Andrews et al., 2009; Yuill et al., 2011) and that nitroxyl-donors (e.g., Angeli’s salt) are effective vasorelaxing agents (Favaloro and Kemp-Harper, 2009; Yuill et al., 2011; Pinkney et al., 2017) in rat mesenteric arteries. An ability to detect both forms of NO would therefore be an advantage in elucidating the relative functional importance of NO• and HNO.

## 5 Conclusion

To conclude, we present a novel, validated method for visualizing NO production in isolated rat mesenteric arteries. This technique has the potential to be applied to arteries from different vascular beds and will further aid research to elucidate the significance of basal vs. stimulated NO release.

## Data Availability

The original contributions presented in the study are included in the article/Supplementary Material, further inquiries can be directed to the corresponding author.

## Supplementary material

Supplementary Movie S1 Time course of Cu_2_FL2E and AF response in a pressurized rat mesenteric artery. The movie shows responses to ACh added at frame 15 and SNAP added at frame 30, equating to a 90 min period. The x- and y-axes were 351 µm and 153 µm respectively, and z-axis was 13.5 µm but the merged average image is shown over time.

Supplementary Movie S2 Time course of DAR-4M and AF response in a pressurized rat mesenteric artery. The movie shows responses to ACh added at frame 15 and SNAP added at frame 30, equating to a 90 min period. The x- and y-axes were 351 µm and 153 µm respectively, and z-axis was 13.5 µm but the merged average image is shown over time.
